# Methods for Similarity-based Virtual Screening

**DOI:** 10.5936/csbj.201302009

**Published:** 2013-03-03

**Authors:** Thomas G. Kristensen, Jesper Nielsen, Christian N. S. Pedersen

**Affiliations:** a Bioinformatics Research Center, Aarhus University, C. F. Møllers Allé 8, DK- 8000 Aarhus C, Denmark; # Now employed by Trifork Gmbh; † Now employed by Google Inc

**Keywords:** Algorithms, Virtual Screening, Fingerprints, SMILES, LINGOsim

## Abstract

Developing new medical drugs is expensive. Among the first steps is a screening process, in which molecules in existing chemical libraries are tested for activity against a given target. This requires a lot of resources and manpower. Therefore it has become common to perform a virtual screening, where computers are used for predicting the activity of very large libraries of molecules, to identify the most promising leads for further laboratory experiments. Since computer simulations generally require fewer resources than physical experimentation this can lower the cost of medical and biological research significantly. In this paper we review practically fast algorithms for screening databases of molecules in order to find molecules that are sufficiently similar to a query molecule.

## Introduction

**Figure 1:**
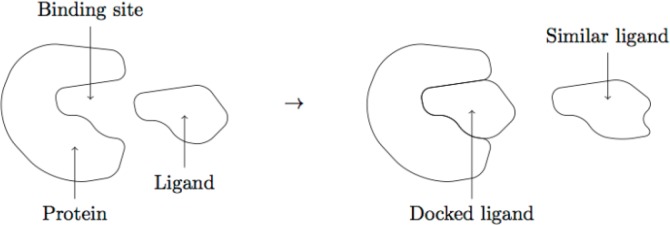
A ligand docking to a protein. Another ligand may dock with the same protein, if it is sufficiently similar.

Proteins are a class of macromolecules that play some of the most important roles in nature. Proteins have functions as catalysts, in signaling and in structural roles. A protein consists of one chain (or multiple chains) of amino acid residues that fold into a more or less rigid structure that has a biological function. A protein that functions as a catalyst will have a certain place, called the *binding site*, where other molecules will *dock* as the protein performs its function. The binding site is usually an indentation or cave in the structure of the protein. A *ligand* is a molecule that docks with another molecule, such as a protein, to perform some function, see Figure 1.

One way to combat a disease is to find a ligand that will dock with a protein important for that disease, and disrupt its normal function. In general one will have a chemical library of molecules that are available for manufacturing. Using computers for predicting the activity of very large libraries of molecules to identify the most promising leads for further laboratory experiments is called *virtual screening*. Simulating the docking between the protein and each ligand on a computer in order search for promising ligands in a library of available molecules requires a lot of computing time and available protein structures.

Instead one may rely on the idea that similar structure leads to similar properties, and predict the properties of a molecule by studying the properties of similar molecules. Hence, if one has identified a ligand that binds to a given target, for example from another medical drug, or observed in nature, one may find other candidate ligands by looking for ligands in a chemical library or database that are similar to the known binder. This similarity- and ligand-based approach to virtual screening works well for the right formalizations of how to represent molecules and quantify their similarity [25]. Due to the size of chemical databases such as PubChem [4] and ChemDB [6], the similarity-baed approach to virtual screening also needs efficient methods for screening a database of molecular representations for molecules that are sufficiently similar to a query molecule. In this paper we review such screening methods for molecules represented as fingerprints or SMILES strings.

## Representing molecules

It is not immediately obvious how to measure the similarity between two molecules. However, some quite simple measures have proven to be surprisingly good when used for virtual screening [14, 22]. For example one might compute a bit-string encoding representative information about the molecules and use the similarity between the bit-strings as a measure of the similarity between the molecules. Such a bit-string for a molecule is denoted a *fingerprint*.

**Figure 2:**
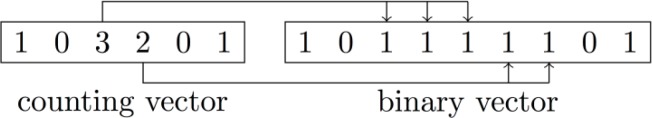
An illustration of converting a counting vector into a binary vector.

There are many ways to compute the actual fingerprints [5]. One general approach is to select a set of features, each of which a molecule may or may not have. Each feature will then correspond to one bit in the fingerprint, and that bit will be set or not, according to whether the given molecule has the feature [26]. Fingerprints of this form will often be quite long, and with many bits set to zero. To use space more efficiently they can be hashed compressed into shorter fingerprints [1, 2, 15]. One might also represent a molecule by a *counting vector* of integers, where each integer counts how many times a certain feature occurs in the molecular. A counting vector allows for a more detailed description of the molecule as a multi-set of features, where a binary fingerprint as introduced above simply describes the molecule as a set of features. However, as counting vectors can easily be converted into binary vectors, for example as illustrated in Figure 2, algorithms for handling binary vectors such as the ones reviewed in this paper are also applicable for counting vectors.

**Figure 3:**
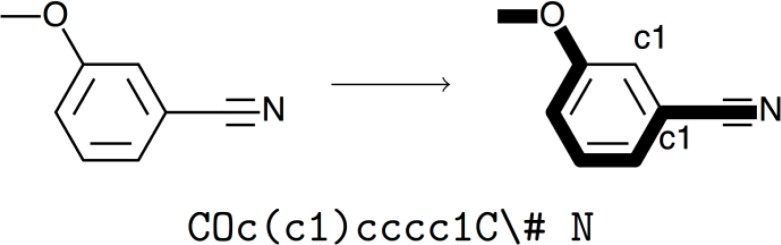
Illustration of a possible SMILES string for 3-cyanoanisole. The primary backbone is highlighted with thick lines. c1 indicates the two points where the ring is merged.

The Simplified Molecular Input Line Entry Specification (SMILES) [23] is a standard way to encode the two dimensional structure of a molecule in a one dimensional string that has a canonical form such that every molecule can be represented by a unique SMILES. The SMILES string is generated by writing a sequence of letters, one for each atom type, marking branches with parentheses and rings with numerical indexes. As an example, consider the visualization of 3-cyanoanisole in Figure 3, which can be represented by the SMILES string “COc(c1)cccc1C\# N”. The main path of the molecule is the string “COcccccC\# N”, the hash mark symbolizing a triple bond. (c1) marks the branch containing just one carbon atom, and the number “1” here and later in the path defines the bond between the two carbon atoms.

Any string of length *n* ≥ *q* will have exactly *n*-*q*+1 substrings of length *q*. In [21], a substring, of length *q*, of a SMILES string is called a LINGO. Thus the SMILES string of a ligand can be viewed as a multi-set of LINGOs, which in [21] is called the LINGO profile of the molecule.

## Similarity between molecules

There are of course several ways to quantify the similarity between two sets (or multi-sets) of features, but the *Tanimoto coefficient* has proven very useful [24, 26]. If *A* and *B* are sets, or multi-sets, of features, then the Tanimoto coefficient, *S*_*T*_ (*A*, *B*), is:

$$S_{T}\left(A,B\right)=\frac{|A\cap B|}{|A\cup B|}.$$

**Figure 4:**
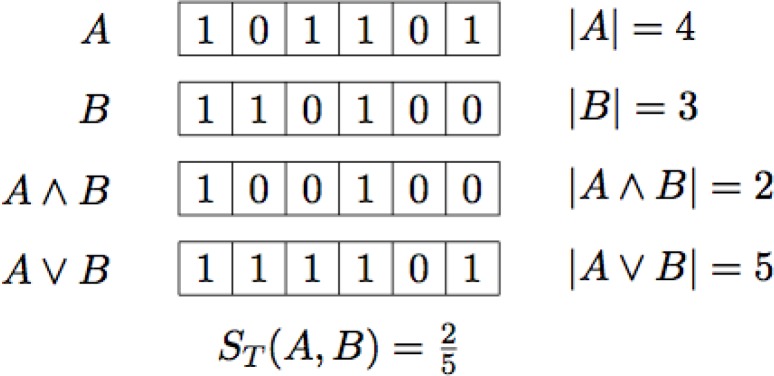
The notation used for bit-strings.

If *A* and *B* are given as two bit-strings, then the Tanimoto coefficient becomes:

$$S_{T}\left(A,B\right)=\frac{|A\wedge B|}{|A\vee B|},$$

where ∧ and ∨ are bitwise logical ‘and’ and logical ‘or’ respectively, and ∣*A*∣ is the number of bits set to one in the bit-string *A*. See Figure 4 for an example.

The Tanimoto coefficient as defined above quantifies the similarity between two bit-strings as a number in the interval [0;1], where 0 says that the two bit-strings have no one-bits in common, and 1 says that the two bit-strings are equal. The coefficient is only defined if there is at least one bit set to one in the two bit-strings (i.e. one feature is shared), which is a very reasonable assumption for molecular fingerprints.

Recall, that the LINGO profile of a molecule is the multi-set of LINGOs in its SMILES string. The similarity between two ligands can thus be measured as the Tanimoto coefficient between their LINGO profiles. This measure is called the LINGOsim between the ligands [21].

One of the major motivations for quantifying molecular similarity is to identify molecules for medical drugs. The problem can be formalized as: We are given a database of representations (for example fingerprints or SMILES) of synthesizable molecules, a query molecule *A*, and a minimal similarity *S*_MIN_. The task is then to find all molecules *B* in the database where *S*_*T*_ (*A*, *B*) ≥ *S*_MIN_. This query can of course be performed by a naive screening of the database, where we examine every fingerprint *A* in the database to compute *S*_*T*_ (*A*, *B*). However, due to the typical size of the database, this is not a desirable approach. In the following sections, we review how to perform such queries more efficiently in practice. We first consider the problem for molecules represented as bit-strings (fingerprints), and secondly, for molecules represented as SMILES.

## Searching for molecules with similar fingerprints

Given a database of *N* fingerprints of length *n*, a query fingerprint *A* (also of length *n*), and a minimal similarity *S*_MIN_. We want to find all molecules *B* in the database where *S*_*T*_ (*A*, *B*) ≥ *S*_MIN_. In [19] it is observed that since ∣*A* ∧ *B*∣ ≤ min(∣*A*∣, ∣*B*∣) and ∣*A* ∨ *B*∣ ≥ max(∣*A*∣, ∣*B*∣), then we can upper-bound the similarity between *A* and *B* by

$$S_{T}\left(A,B\right)=\frac{|A\wedge B|}{|A\vee B|}\leq \frac{\min\left(|A|,|B|\right)}{\max\left(|A|,|B|\right)}=COUNT-MAX\left(A,B\right).$$

Such an upper-bound can be used to make queries faster than a simple linear search by sorting the fingerprints *B* in the database into bins depending on their counts of one-bits ∣*B*∣. When a query is performed we can compute which bins has a COUNT-MAX (*A*, *B*) ≥ *S*_MIN_ and only examine the fingerprints in those bins, i.e. only compute *S*_*T*_ (*A*, *B*) for the fingerprints *B* in those bins.

In a later paper [3], it is suggested to use a filter based on XOR signatures to improve this pruning even further. The idea is to first split the fingerprints into *k* equal-sized fragments such that *A* = *A*_1_ *A*_2_···*A*_*k*_ and *B* = *B*_1_ *B*_2_···*B*_*k*_ and then compute XOR-signatures, *a* and *b* of *A* and *B*, as *a* = *A*_1_ ⊕ *A*_2_ ⊕···⊕ *A*_*k*_, and *b* = *B*_1_ ⊕ *B*_2_ ⊕···⊕ *B*_*k*._ Since ∣*A* ⊕ *B*∣ = (∣*A*∣ + ∣*B*∣ - ∣*A*⊕*B*∣) / 2 and ∣*A* ⊕ *B*∣ = (∣*A*∣ + ∣*B*∣ + ∣*A*⊕*B*∣) / 2 and we can lower-bound the size of the XOR of the fingerprints by the size of the XOR of the signatures, i.e. ∣*A* ⊕ *B*∣ ≥ ∣*a* ⊕ *b*∣, then we can bound the similarity as

$$S_{T}\left(A,B\right)=\frac{|A|+|B|-|A⊕B|}{|A|+|B|+|A⊕B|}\leq \frac{|A|+|B|-|a⊕b|}{|A|+|B|+|a⊕b|}=\text{XOR-MAX}\left(A,B\right).$$

Since the XOR signatures *a* and *b* are shorter than the original fingerprints *A* and *B*, we can compute *a*⊕*b* faster than *A*⊕*B*. This is used as a filter, where the fingerprints *B* in the database *DB* are still stored in bins depending on ∣*B*∣, but the signature of each fingerprint is stored with it, and the final *S*_*T*_ (*A*, *B*) is only computed for fingerprints where XOR-MAX (*A*, *B*) ≥ *S*_MIN_.

In [16] it is suggested to store the database *DB* as a trie [7]. The key observation is that by walking down the trie one can bound the similarity between the query fingerprint *B* and any fingerprint *B* in a leaf below the current node in the trie. Consider a node at a level *d* in a trie. Let *A*_HEAD_ be the first *d* bits of the query fingerprint *A*, and *A*_TAIL_ be the remaining *n*^–^*d* bits. Similarly for an arbitrary database fingerprint *B* below the node. We may now observe that

$$|A\wedge B|\leq |A_{\text{HEAD}}\wedge B_{\text{HEAD}}|+|A_{\text{TAIL}}|,$$

$$|A\vee B|\geq |A_{\text{HEAD}}\vee B_{\text{HEAD}}|+|A_{\text{TAIL}}|,$$

and hence

$$S_{T}\left(A,B\right)=\frac{|A\wedge B|}{|A\vee B|}\leq \frac{|A_{\text{HEAD}}\wedge B_{\text{HEAD}}|+|A_{\text{TAIL}}|}{|A_{\text{HEAD}}\vee B_{\text{HEAD}}|+|A_{\text{TAIL}}|}=\text{TRIE-MAX}\left(A,B\right).$$

Thus, like above, we need only visit the children of the current node if TRIE-MAX (*A*, *B*) ≥ *S*_MIN_. A trie easily takes up a lot of memory, so in [17] the trie is compressed by collapsing long runs of zero-bits into one node. This works well, because molecular fingerprints tend to be sparse.

In [11], we present the kD-grid, which is a data structure for supporting fast queries in practice building upon the ideas outlined above in the sense that it corresponds to the approaches in [17] and [19] for certain choices of the parameter *k*.

**Figure 5:**
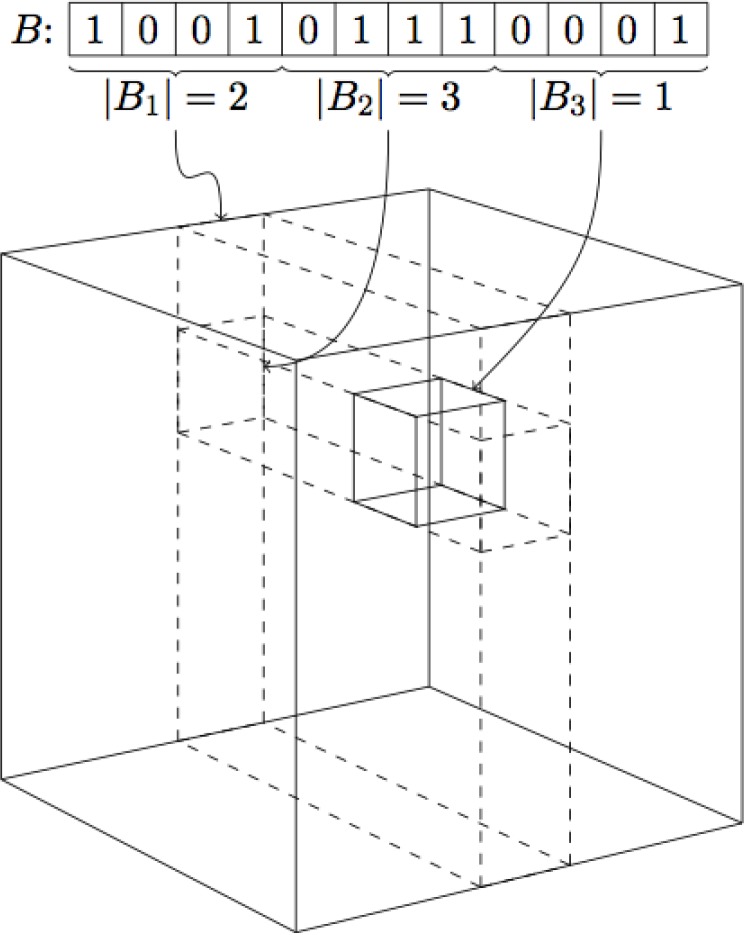
An example of a fingerprint being stored in a 3D-grid.

In the kD-grid, we split all fingerprints into *k* equal-sized fragments such that *A* = *A*_1_ *A*_2_···*A*_*k*_ and *B* = *B*_1_ *B*_2_···*B*_*k*_, and all database fingerprints *B* in the database *DB* are placed into bins in a *k* dimensional grid, based on the bit counts of the fragments ∣*B*_*j*_∣. Figure 5 illustrates how a fingerprint is stored in a 3D-grid. Like above, we can compute bounds on the similarity between a query fingerprint *A* and the database fingerprints in any of these bins

$$S_{T}\left(A,B\right)=\frac{|A\wedge B|}{|A\vee B|}\leq \frac{\Sigma_{i=1}^{k}\min\{|A_{i}|,|B_{i}|\}}{\Sigma_{i=1}^{k}\max\{|A_{i}|,|B_{i}|\}}=\text{GRID-MAX}\left(A,B\right).$$

**Figure 6:**
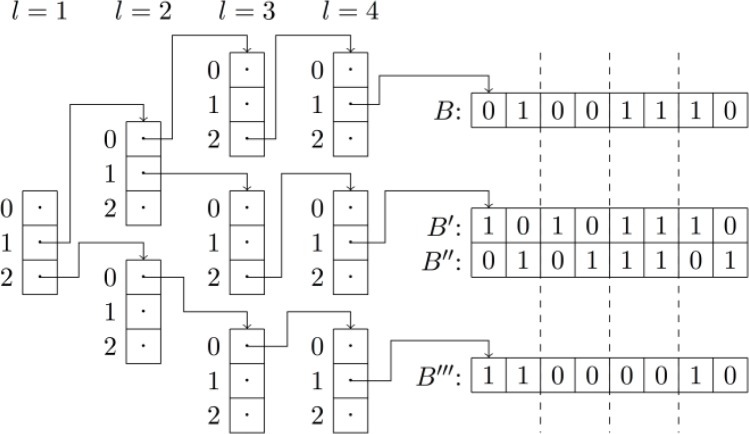
An example of four fingerprints being stored in a tree representing a 4D-grid.

In practice we implement the grid as a tree with *k* levels and leaves of degree *n*/*k*, but with branches without leaf fingerprints pruned, see Figure 6. When looking up a query, we walk down the tree and can compute bounds on all sub-branches. Assume we are visiting a node at level *l* in the tree. The bound is then

$$S_{T}\left(A,B\right)\leq \frac{\Sigma_{i=1}^{k}\min\{|A_{i}|,|B_{i}|\}+\Sigma_{i=l+1}^{k}|A_{i}|}{\Sigma_{i=1}^{k}\max\{|A_{i}|,|B_{i}|\}+\Sigma_{i=l+1}^{k}|A_{i}|}$$

Note that if we set *k* = 1 this corresponds to the approach of [19] and if we set *k* = *n* this becomes the trie of [17].

**Figure 7:**
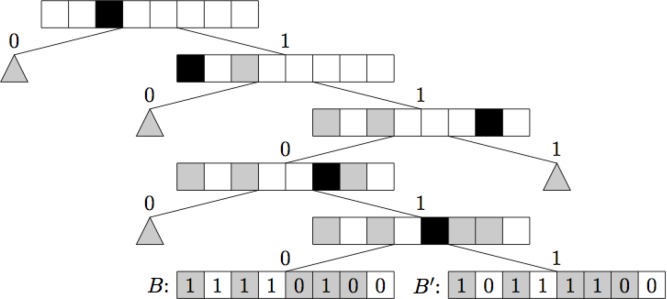
Example of a singlebit tree. The black squares denote the bits on which the data is split; while the gray squares denote bits we have information about from further up the tree.

Naively one would store the fingerprints in each bin in a simple list, but one can do better. In [12] we present two alternative data structures for representing the bins. The first is the *singlebit tree*. The fingerprints for a given bin will be stored in the leaves of a tree, while the internal nodes each store the index of a bit. Fingerprints with the indexed bit clear will be stored in the left sub-tree of the given node, and fingerprints with the indexed bit set will be stored in the right sub-tree, see Figure 7. Thus it is similar to a trie, except the bits in the bitstring can be examined in any arbitrary order, instead of left-to-right, as they are in a trie. Also, since we know what bucket the singlebit tree is sitting in we have information about the number of set bits for all fingerprints in the entire tree, which allows us to derive tighter bounds than those of [17]. Let *M*_ij_ be the count of positions where *A* has an *i*-bit and *B* has a *j*-bit. For example *M*_10_ is the number of positions where *A* has a one and *B* has a zero. Walking down a singlebit tree we will obtain partial knowledge of *M*_ij_, as we compare the bits in the nodes of the tree with those in *A*. Let the number of positions we have knowledge about, and where *A* has an *i*-bit and *B* has a *j*-bit, be *m*_*ij*_ and the unknown difference *m*_*ij*_ and *M*_*ij*_ be *u*_*ij*_, i.e. *M*_*ij*_ *= m*_*ij*_ + *u*_*ij*_^.^ Now we can bound the Tanimoto coefficient by

$$S_{T}\left(A,B\right)=\frac{M_{11}}{M_{01}+M_{10}+M_{11}}\leq \frac{\min\{|A|-m_{10},|B|-m_{01}\}}{\max\{|A|+m_{01},|B|+m_{10}\}}$$

and only visit subtrees where the leaves may be sufficiently similar to the query fingerprint.

**Figure 8:**
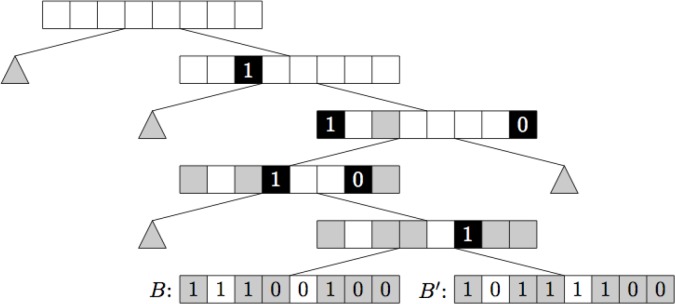
Example of a multibit tree. The black squares denote the match bits, while the gray squares denote bits that are match bits further up the tree.

Improving upon this we also suggested the *multibit tree*. The multibit tree is similar to the singlebit tree, but stores several bits in each internal node instead of only one. This means that we can no longer split the children of a node based on whether they have a one or a zero, thus the semantics needs to change somewhat. For each node in the tree store a list of bit positions, along with a Boolean value. These bits we call the match bits. The match bits of a node are exactly those bits for which all the children of the node have the same value and that is not a match bit further up the tree see Figure 8. Walking down a multibit tree we again gain partial knowledge about the leaves of the tree and exactly the same bound as that of the singlebit tree may be used.

**Figure 9:**
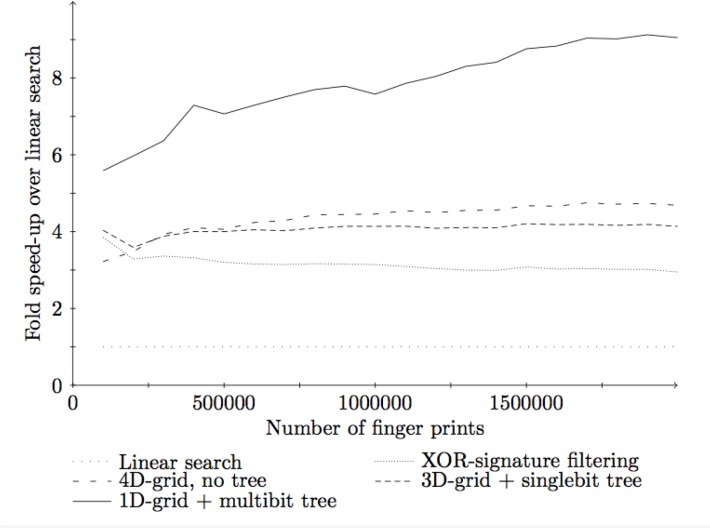
The result of an experiment that compares the running time versus database size for our algorithms, the previous best algorithm (XOR-signature filtering [3]), and a naive linear search. Fingerprints were generated using the CDK fingerprint generator [18] with a standard fingerprint size of 1024. We have performed tests on data set, containing from 100,000 to 2,000,000 fingerprints in 100,000 increments. For each data set size, the entire data structure was created. Next, the first 100 fingerprints in the database are used for queries. Each experiment is performed 100 times, and the average query time is presented as the speed-up compared to the naive linear search. All experiments are performed with a SMIN of 0.9. For each kD-grid, the *k* (1, 2, 3, or 4) that gave the best results was chosen.

How best to build the singlebit and multibit trees are not obvious. The algorithm we used in our implementation is to split the dataset recursively into smaller and smaller subsets. For each set of fingerprints we choose the bit that splits the tree into two subsets that are maximally close to having the same size. The set is then split into two subsets based on whether that bit is set or not for each fingerprint. The reasoning is to attempt to obtain a tree that is as well balanced as possible. Theoretically it is not clear that this is the right way to build the trees, but in practice the methods perform well, as illustrated by the experimental results reported in Figure 9. The SymDex method [20] is a recent method that is reported to perform even better.

## Searching for molecules using LINGOsim

The LINGOsim [21] similarity measure is attractive because it only relies on the SMILES description of the molecule, and it has proven to be competitive with more computationally expensive methods for predicting ligand properties, despite its simplicity [8]. An efficient method for computing the LINGOsim, using a finete state machine, is presented in [8]. Given a query SMILES string *A* and a database, they suggest building a finite state machine from *A* to be able to quickly compare it against any other SMILES string.

Recall, that the LINGO profile of a molecule is the multi-set of LINGOs in its SMILES string, and that the LINGOsim similarity between two ligands is the Tanimoto coefficient between their LINGO profiles. The algorithm described in [8] starts by building a trie from all length *q* substrings of *A*. This trie is then converted into a finite state machine, where states correspond to length *q* strings, and substrings in *A* are accepting states. By running this state machine on another SMILES string *B*, the size of the intersection of *A* and *B* can found efficiently, and since ∣A∣+∣B∣=∣A∩B∣+∣A∪B∣, we can also find the size of the union of *A* and *B* efficiently. With the sizes of the intersection and union in hand, the LINGOsim is straightforward to obtain.

A parallel algorithm is suggested in [9]. Their first observation is that since a character in a computer normally uses eight bits, and it is shown in both [21] and [8] that the optimal length of LINGOs is *q* = 4, then a LINGO can be stored in a 32-bit computer word. For each molecule they explicitly store a sorted list of all LINGOs in the SMILES string of the molecule, along with a count of how many times each LINGO occur in the molecule. This allows the intersection size of LINGOs between two molecules to be computed by iterating over the two LINGO lists simultaneously, similar to the merge of a merge-sort. As above the intersection size is enough to compute the LINGOsim. Parallelization is achieved by processing several molecules at the same time and the paper presents implementations for both CPUs and GPUs. They name their algorithm SIML for Single-Instruction Multiple-LINGO.

**Figure 10:**
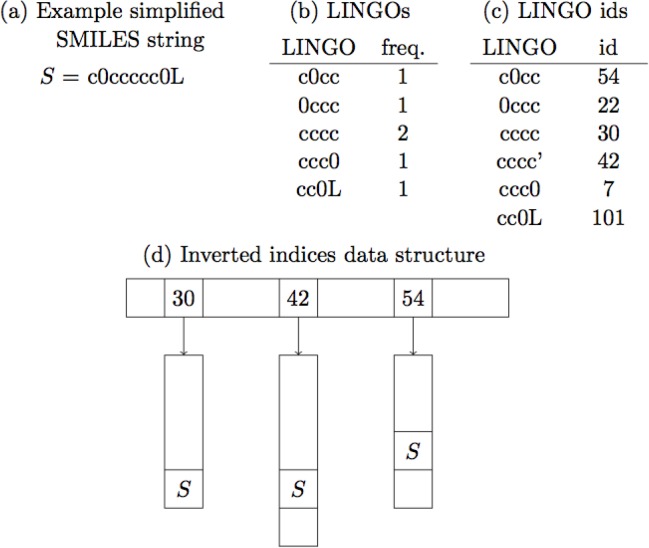
From SMILES strings to inverted index. (a) SMILES string simpli- fied for LINGOsim. (b) LINGOs of example SMILES string. (c) The LINGOs are given ids, with multiple occurrences given unique ids. (d) A reference to the SMILES string *S* is stored for all the ids of the LINGOs in *S*.

In [13], we suggest using an inverted index to compute the LINGO intersection size between a query SMILES string *A* and the entire database quickly. First a preprocessing step is necessary, where each LINGO in the database is given an integer number, such that the first occurrence of a given LINGO in a SMILES string is given a unique id, the second occurrence another one, and so on. This step reduces the problem from multi-sets of LINGOs to ordinary sets of ids. Next we store the database in an inverted index [10], that is, instead of storing a list of LINGOs or ids for each database SMILES string, we store a list of SMILES strings for each LINGO id. See Figure 10. Inverted index algorithms also have been applied to speed up fingerprint similarity searches [16].

Now we can compute the LINGO intersection size for the entire database the following way: Create a counter for each database SMILES string. For each LINGO in the query *A* increase the counter of all database SMILES strings found on the list of that LINGO. When all LINGOs have been processed the counters contain the LINGO intersection sizes, from which the LINGOsims can be computed. This is fast because we only need to visit relevant LINGOs in the database, as opposed to the above methods that always query the entire database. Like above we can parallelize this by processing several molecules at the same time.

**Figure 11:**
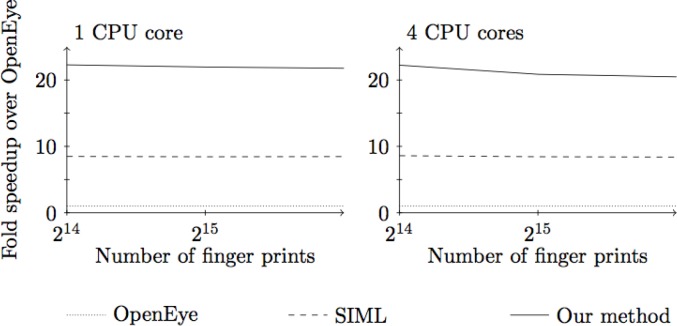
Comparison of our implementation against that of OpenEye and SIML [9], for one and four CPU cores.

For benchmarking we use a method similar to that of [9], computing the LINGOsim similarity of all pairs of fingerprints in the database. We compare our implementation against SIML [9] and the commercial OpenEye implementation. The details of the experiment are described in [13] and the results are summarized in Figure 11.

## Conclusions

In this paper, we have reviewed computationally efficient methods for solving the problem of identifying all molecules stored in database that have a certain similarity to a query molecule. We have considered to problem when molecules were represented by bit-strings, and when molecules were represented by SMILES string. In both cases, the similarity measure used has been the Tanimoto coefficient. The growing size of chemical databases implies a growing need for solutions to this problem that are efficient in practice.

An area for improvement that we have not considered in details is memory usage. Our data structures consume a lot of memory. To store very large molecule databases it might be relevant to create an I/O efficient implementation that stores the data structures on disk in way that can be processed efficiently without reading the entire structure into memory.
